# Emotional Change-Associated T Cell Mobilization at the Early Stage of a Mouse Model of Multiple Sclerosis

**DOI:** 10.3389/fimmu.2013.00400

**Published:** 2013-11-21

**Authors:** Giuseppa Piras, Lorenza Rattazzi, Adam McDermott, Robert Deacon, Fulvio D’Acquisto

**Affiliations:** ^1^William Harvey Research Institute, Barts and The London School of Medicine, Queen Mary University of London, London, UK; ^2^Department of Experimental Psychology, University of Oxford, Oxford, UK

**Keywords:** T cells, multiple sclerosis, immunomodulation, mood disorders, behavior

## Abstract

Autoimmune diseases like multiple sclerosis (MS) are known to be associated with debilitating emotional disorders that manifest long before the flaring of motor dysfunctions. Given the emerging role of T cells in controlling both emotions and autoimmunity, in this study we explored possible correlation between T cell activation and changes in emotional behavior in a mouse model of MS. Our results showed a significant increase in blood circulating T cells as soon as at day 4 post immunization. This lymphocytosis remained stable with time and preceded the infiltration of T cell in the CNS. The kinetic of T cell entry in the blood matched the kinetic of changes in behavior measured using the open field test. Treatment with glatiramer acetate, a well-known immunomodulatory drug for MS, suppressed behavioral changes while retaining the T cells in the draining lymph nodes. Together these results provide evidence of a positive correlation between the emigration of T cells in circulation and changes in emotions during chronic inflammatory diseases. The validation of these findings in the clinic might help to better understand the cause of the emotional and psychological burden of patients suffering MS or other autoimmune diseases. Most importantly our study suggests novel therapeutic venues for the treatment of the emotional changes associated with autoimmunity.

## Introduction

A wealth of studies in the literature has indicated a significant increase in emotional changes in patients suffering from multiple sclerosis (MS) (1–3) as well as from other autoimmune diseases (4). Major depression (5–7), bipolar depression (2, 8), anxiety (9–11), alcohol abuse (12, 13), and other substance abuses (14) are all at an increased prevalence in MS population. These emotional dysfunctions are not simply a reactive psychological response to the impact of this pathology on the patient’s life style and have been correlated with the development of MS and other autoimmune diseases. Indeed, the depression and anxiety rates are higher in MS than in those patients experiencing other chronic diseases (15). It is in fact estimated that between 40 and 50% of patients with MS will experience a type of depression within their lifetime. As consequence of this, MS patients show a higher rate of suicides when compared to a normal population with most occurring within 5 years of diagnosis (16, 17).

One of the most unexpected aspects of the correlation between emotional disorders and MS is their association in time. Recent evidence suggests that depression usually presents before the onset of MS symptoms or even before diagnosis (9, 18) and with over a third of MS patients having a family history of depression (19, 20). Indeed, looking at MS patient blogs^1^^,^^2^ as well as at systematic epidemiological studies, it is clear that patients often lament of having suffered panic attack or anxiety over limited period of time. In other words, patients suffered from unexpected attacks of anxiety that did not necessarily correlated with any manifestation of the disease. In other cases, it seems that these “bouts” of anxiety and panic attack precede or follow the same pattern of MS. Most intriguingly, MS and mental disorders like depression show a large degree of similarities. Indeed, both can provoke cognitive impairment, muscle weakness, or tiredness (21–23).

Previous studies, summarized in Table 1, addressed the behavioral modifications occurring in mouse models of MS, the experimental autoimmune encephalomyelitis (EAE). These studies reported either no changes (24) or an inverse correlation between social exploration and the rise of inflammatory mediators including IL-1, TNF-α, and PGE_2_ (25). Conversely, Peruga et al. demonstrated that mice immunized with a suboptimal dose of MOG_35–55_ (50 μg) showed the manifestation of motor impairment at day 60 after immunization and had an increase anxiety-like behavior that correlated with an increase in the level of TNF-α and with neuronal loss in the hippocampus (26). This was also associated with a doubled depressive-like behavior in the learned helplessness paradigm. In a more recent study, Haji et al. assessed the behavior of mice subjected to EAE before locomotor defects started to show (27). Their results suggested firstly that high anxiety indexes in EAE mice precede the appearance of motor defects and secondly that TNF-α has a pivotal role in the high anxiety response because of the ability of this cytokine to cause striatum inflammation and microglia activation. In addition, intracerebroventricular administration of etanercept, an inhibitor of TNF-α signaling, resulted in anxiolytic-like effects in EAE mice.

**Table 1:** **Previous studies characterizing behavioral changes in mouse models of EAE**.

	Pollak et al. (25)	Peruga et al. (26)	Rodrigues et al. (24)	Haji et al. (27)	Acharjee et al. (28)
Animals	Female SJL/J mice	Female C57BL/6 mice	Female C57BL/6 mice	Female C57BL/6 mice	Female C57BL/6 mice
EAE protocol	150 μg of PLP_139–151_ 15–20 × 10^6^ activated lymph node cells i.p.	50 μg of MOG_35–55_ 100 ng of PTX i.p.	100 μg of MOG_35–55_ 300 ng of PTX i.p.	200 μg of MOG_35–55_ 500 ng of PTX i.p.	100 μg of MOG_35–55_ 800 ng of PTX i.p.
Onset of motor deficits (days)	Not specified	Signs of tail weakness at 60 dpi	Clinical signs of disease at 11 dpi	Expected at 10–11 dpi [according to Ref. (29)]	Limp tails at 9–13 dpi
Behavioral parameters/paradigms	Food and sucrose intake; social exploration	Open field; rotarod; light/dark box; startle response and pre-pulse inhibition; learned helplessness paradigm	Elevated plus maze; inhibitory avoidance task; object recognition task	Open field; elevated plus maze	Open field; elevated plus maze; forced swim test; tail suspension; sociability test; fear conditioning
Cytokine levels	IL-1β expression/level (RT-PCR/ELISA) and TNF-α expression (RT-PCR); PGE2 production (RIA assay); brain (cerebellum, hypothalamus, hippocampus, brain stem)	IL-6 and TNF-α expression (RT-PCR); brain (hippocampus); 15, 29, 41, 59 dpi	–	TNF-α levels (ELISA); Brain (striatum); 10 dpi	IL-1β and TNF-α expression (RT-PCR); brain (hippocampus, hypothalamus, amygdala) 7 dpi
Main results	Transient sickness behavior episodes associated with EAE attacks; Increased pro-inflammatory cytokine levels before the onset of motor impairment; decrease in pro-inflammatory cytokines at the peak of the neurological symptoms	Anxiety- and depression-like behavior before the occurrence of motor deficits; Increased TNF-α and neuronal loss in the hippocampus	No differences in anxiety-like behavior and memory in animals induced with EAE	Anxiety-like behavior before the occurrence of motor deficits; Increased TNF-α levels and activated microglia in the striatum	Anxiety- and depression-like behavior, memory loss and conditioned learning deficits in early stage of EAE; elevated levels of IL-1β and TNF-α in the hypothalamus and increased basal plasma corticosterone levels

All these studies focused on the biochemical and cellular changes occurring in the CNS while very little has been explored in terms of possible changes occurring in the periphery such as in the blood. Indeed, a great deal of studies, including our own in RAG-1^−/−^ (30), have shown that T cells plays a pivotal role in regulating emotion in mice (31–34) as well as in humans (35, 36) besides being the main drive of autoimmune diseases.

In this study we set to investigate the correlation between emotional changes and T cell activation during the very early stages of the EAE. Consistent with the already published experimental and clinical studies mentioned before, our results confirmed that emotional changes occur long before the manifestation of motor dysfunction and within the first days after the immunization. In addition, we provide evidence of a direct correlation between changes in behavior and the time-dependent activation and expansion of T cells, thus confirming a very tight crosstalk between immunity and mental health during the development of chronic inflammatory diseases.

## Materials and Methods

### Mice

We used 6-week-old male mice for all the experiments. Mice were housed in groups of six per cage under specific-pathogen-free conditions and with free access to food and water. Mice were housed for at least 7 days prior to testing. Wild type C57BL/6 mice purchased from Charles River. All experiments were performed during the light phase of the light-dark cycle and no more than two tests per day were performed. All tests were conducted under license from the Home Office and according to the UK Animals (Scientific Procedures) Act, 1986.

### MOG_35–55_-induced experimental autoimmune encephalomyelitis

This model of autoimmunity is mainly driven by T cells and has been extensively used to investigate the early events that characterized the development of MS including the activation of the immune response that precedes the neuronal damage caused by inflammatory cells (37). Male C57BL/6 mice received an intradermal injection of MOG_35–55_ (300 μg) emulsified in Complete Freund’s adjuvant (CFA) and two doses of pertussis toxin (PTX) at day zero and day 2 as previously reported (38). The MOG_35–55_/CFA emulsion was prepared by dissolving 300 μg of MOG_35–55_ peptide (MEVGWYRSPFSRVVHLYRNGK, synthesized by Cambridge Research Biochemicals, Cleveland, UK) in 150 μl of PBS and then mixed with 150 μl of CFA (Complete Freud’s Adjuvant, Sigma-Aldrich). The resulting suspension was emulsified using a high-pressure polytron homogenizer. The severity of the disease was scored on a scale of 0–6 with 0 = no neurological signs, 1 = tail weakness, 2 = tail paralysis, 3 = loss of righting reflex (the mouse can no longer right themselves after being laid on their back), 4 = hind leg paralysis, 5 = quadriplegia, and 6 = death. In some experiments, mice were immunized with CFA only or with the antigenic ovalbumin peptide OVA_323–339_ (100 μg) and received the two doses of PTX at day 0 and 2. For the treatment with glatiramer acetate (GA; Poly Ala, Glu, Lys, Tyr [6:2:5:1], Sigma), mice were subcutaneously immunized with GA (150 μg/100 μl of PBS) every day for 7 days before the immunization with MOG_35–55_/CFA. Control mice were administered the same volume of PBS vehicle.

### Leukocytes isolation from central nervous system

Vertebral columns were dissected from the lumbar to the cervical region and washed several times in PBS to remove blood trace. Spinal cords were extracted by hydro pressure in the spinal canal by using a 2-ml syringe and 19-gage needle. Subsequently, tissues were torn apart in sterile PBS by mechanical pressure through a 70-μm mesh cell strainer (Falcon). Mononuclear cells and lymphocytes were isolated by density gradient centrifugation in Percoll (GE Healthcare). In detail, cells were pelleted at 400 g for 5 min and suspended in a 30% Percoll solution. The 30% Percoll solution was carefully layered onto a 70% Percoll solution in a ratio 2:1 and centrifuged at 500 *g* for 30 min. In this density gradient mononuclear cells sediment at the interface between 30 and 70% Percoll layers. About 2–3 ml of interface solution was collected only after the fatty layer at the top of the centrifuge tube was carefully removed. The purified mononuclear cells were washed twice in RPMI supplemented with 100 U/ml of penicillin and streptomycin and 10% of FCS (Invitrogen).

### Flow cytometric analysis

Lymphocytes were stained in 100 μl of FACS buffer (PBS containing 5% FCS and 0.02% of NaN_3_) containing the following antibodies: anti-CD3 PE-Cy5 (clone 145-2C11, eBioscience), anti-CD4 FITC (clone GK 1.5, eBioscience), anti-CD8 PE (clone 53-6.7, eBioscience) as previously reported (39). Cells were labeled with the appropriate concentration of conjugated antibodies for 1 h at 4°C as previously described. Samples were acquired with FACSCalibur and analyzed using FlowJo™ software (Tree Star, Inc., Oregon Corporation). Peripheral blood leukocytes were collected at different time points after immunization. Briefly, blood samples were collected by intracardiac puncture performed under anesthesia in syringes containing sodium citrate 3.2% (w/v). Cells were pellet at 300 g and resuspended in FACS buffer containing 1:500 Fc blocking antibody (anti-mouse CD16/32) and then stained with anti-CD3 (clone 145-2C11). Red blood cells were lysed with RBC Lysis Buffer according to the manufacturer’s instruction (eBioscience).

### Plasma cytokine measurement

Blood was collected by intracardiac puncture performed under anesthesia. Plasma was obtained from the clotted blood by centrifugation (8000 rpm, 5 min) and stored at −80°C before the assay. Cytokine levels in the same samples were measured (dil. 1:500) using Mouse Th1/Th2/Th17/Th22 16plex Kit FlowCytomix and according to the manufacturer’s instructions (eBioscience).

### Open field activity test

The open field is a test commonly used to assess locomotor, exploratory, and anxiety-like behavior in laboratory animals. It is based on the conflict between the spontaneous aversion that rodents have of the central area of a novel or brightly lit open field versus their desire to explore new environments (40). The test was performed as previously described with some modifications (41). The open field consisted of a white PVC arena (i.e., a plastic rectangular container size 50 cm × 30 cm) divided into 10 cm × 10 cm squares (*n* = 15). Mice were brought into the experimental room 15 min before testing. Each mouse was placed in one of the corner squares facing the wall. A mouse was observed and recorded for 5 min. The total number of squares crossed, latency to the first rear and the total number of rears were assessed. After each test the arena was cleaned with water to attenuate and homogenize olfactory traces.

### Data analysis

Pairwise comparisons were made by *t*-test and comparisons of more than two groups were analyzed using one-way ANOVA. The differences in behavior between control and immunized mice were determined using two-way repeated measure ANOVA and day-by-day Bonferroni post-test. The results were expressed as mean ± SEM. Fit linear regressions and 95% confidence bands to the means of parameters over time were calculated by Prism (GraphPad software).

## Results

### Phases of disease in MOG_35–55_-induced EAE

**Figure 1:**
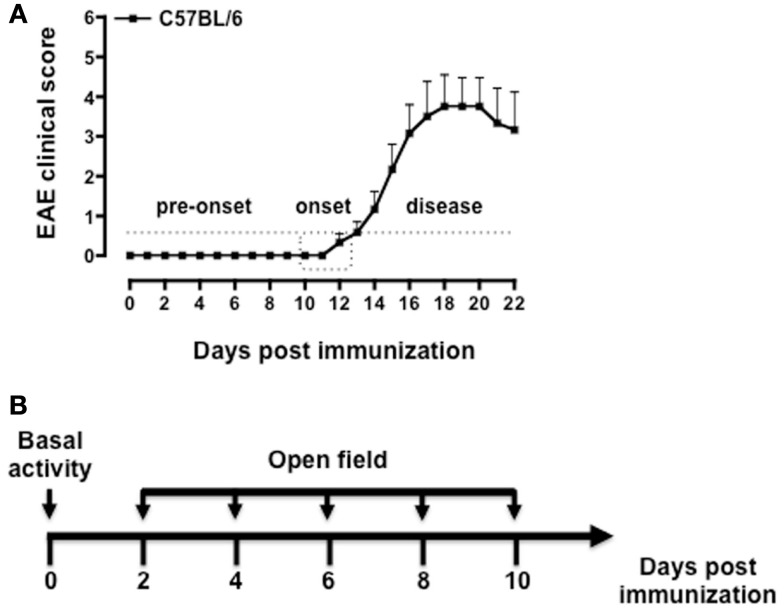
**Phases of the disease in MOG_35–55_-induced EAE and behavioral assessment protocol**. The graph in **(A)** shows a typical pattern of development of the EAE clinical score with the three main phases of the disease while the scheme in **(B)** shows a schematic of the behavioral test protocol used in the study. The results in **(A)** are from a single experiment with *n* = 10 mice and are representative of *N* = 5–6 separate experiments.

Immunization of C57BL/6 mice with MOG_35–55_/CFA causes a neurodegenerative inflammatory disease that resembles the primary progressive form of MS (42, 43). Although the manifestations of the disease are not always synchronous in all the mice, it is possible to distinguish three main phases: the pre-onset, the onset, and the disease phase (Figure 1A). During the pre-onset (day 0–10), mice do not show any visible motor defects while behavioral changes are readily visible since day 2 post immunization. At the onset of the disease (day 10–12), mice develop a weak or flaccid tail and start to show signs of motor dysfunction. During the disease phase (day 12–22) mice progressively loose the ability to move the hind legs and a significant weight loss (up to 10%) occurs. Mice were tested starting from day 2 (before PTX injection) and every other day till day 10, i.e., before the occurrence of any motor defect (Figure 1B).

### Changes in behavior in the open field test

The open field test has been previously used in the majority of studies assessing anxiety behavior during EAE (see Table 1). We used this test as read-out system for the behavioral changes at the early stages of the EAE. The convenience of this test is that it provides easy and simultaneous measure of multiple parameters including locomotion, exploration, and anxiety.

**Figure 2:**
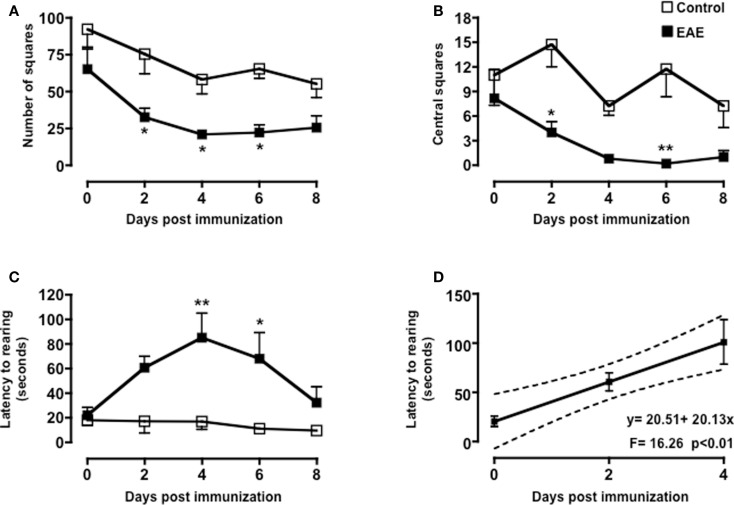
**Behavior of MOG_35–55_-immunized mice in the open field test**. The graphs show the total number of squares crossed **(A)**, number of central squares entries **(B)**, latency to rearing, and the relative linear regression **(C,D)** of control or MOG_35–55_-immunized mice assessed during a 5-min test. Values are expressed as mean ± SEM for six to eight mice and are representative of *N* = 5–6 separate experiments. *p < 0.05, **p < 0.01 indicate significant values compared to control mice.

As shown in Figure 2, both MOG_35–55_/CFA immunized mice and PBS-treated control group showed a gradual and time-dependent decrease in locomotor activity (between 50 and 35%, respectively) that became almost stable from day 4 onward (*p* < 0.05; Figure 2A). The number of passages through the central square is considered a measure of anxiety and exploratory activity in this test. Control mice showed a variable but overall stable number of central square visits throughout the 8-days of testing. In contrast, immunized mice showed a significant reduction by day 2 and a further decrease at day 4. This value also remained constant for the next 4 days (*p* < 0.01; Figure 2B).

**Figure A1:**
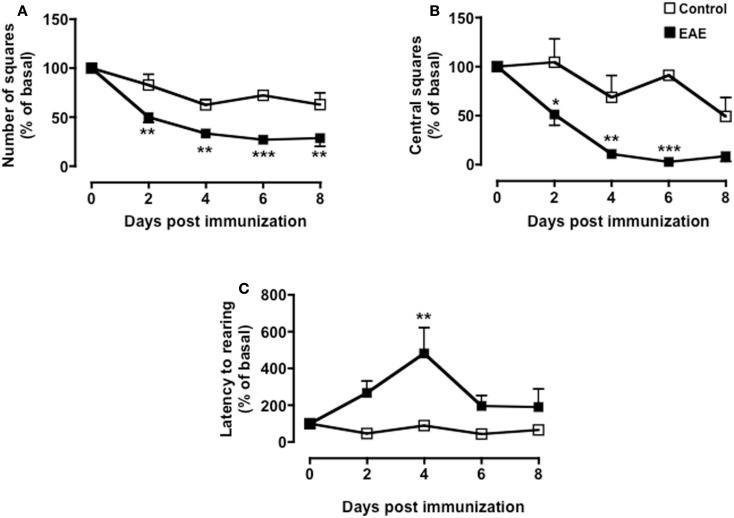
**Behavior of MOG_35–55_-immunized mice in the open field test**. The graphs show the percentage of total number of squares crossed **(A)**, number of central squares entries **(B)**, and latency to rearing **(C)** of control or MOG_35–55_-immunized mice compared to their baseline levels assessed during a 5-min test. Values are expressed as mean percentage ± SEM for six to eight mice and are representative of *N* = 5–6 separate experiments. *p < 0.05, **p < 0.01, ***p < 0.001 indicate significant values compared to control mice.

Rearing in mice and other rodent is an emotional and protective response to the stress of a new environment; this is a typical vertical activity that consists in the standing completely erect on the hind legs. This “risk-assessment” behavior indicates that the animal is hesitant to move from its present location to a new position. In the open field test, the latency to the first rear is considered a measure of depression and associated anxiety (40, 44). Control mice did not show any significant changes in the number of rearing (data not shown) or in the latency to the first rear throughout the time of the experiment. Conversely, immunized mice showed a steep increase in latency to rearing until day 4 and then a decline to almost basal level from day 8 (Figure 2C). When we compared the fold changes versus baseline values of all the parameters we have measured (Figure A1 in Appendix), the latency to rearing showed the highest fold change (about fivefold). Most interestingly, this followed a linear correlation from day 0 to 4 with a slope that was significantly different from zero [*b* = 20.51, *F*(1,15) = 16.26, *p* < 0.01; Figure 2D].

### Behavioral changes at the early stage of EAE mirror the expansion and mobilization of T cells

**Figure 3:**
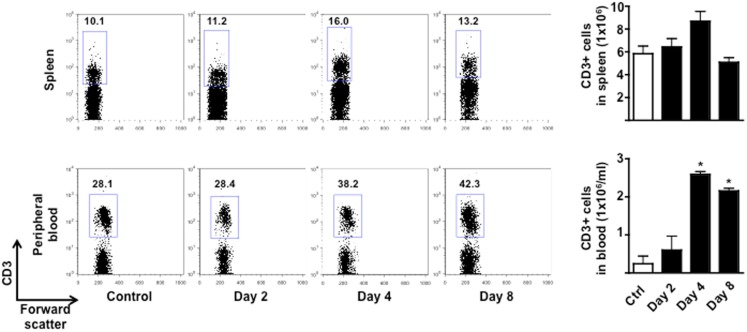
**Expansion and mobilization of T cells during the early stages of MOG_35–55_-induced EAE**. The dot plots show the percentages of CD3^+^ T cells while the bar graphs show the comparison of the total number of CD3^+^ T cells in spleen (*top panels*) or peripheral blood (*bottom panels*) of control or MOG_35–55_-immunized mice. Values are expressed as mean ± SEM for three to four mice. *p < 0.05 indicates significant values compared to control mice.

**Figure A2:**
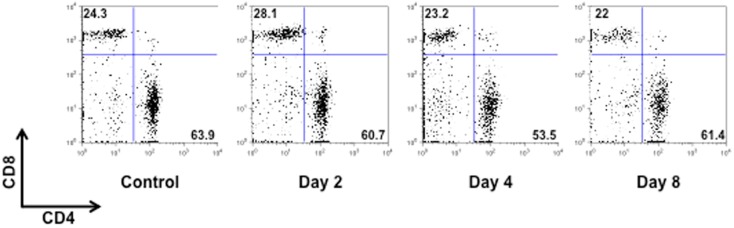
**CD4/CD8 T cell profile of MOG_35–55_-immunized mice**. The dot plots show the percentages of CD4^+^ and CD8^+^ T cells in the spleen of control or MOG_35–55_-immunized mice sacrificed at the indicated time points. The results are representative of *n* = 6–8 mice.

To test if the changes in behavior observed in immunized mice were correlated to early cellular and molecular events that are important for the development of EAE, we sacrificed mice at day 0, 2 and 4, and 8 and collected peripheral blood and spleens. As shown in Figure 3, the total number of CD3^+^ cells in the spleen peaked at day 4 and then returned to basal level at day 8. No changes in the percentages of CD4 or CD8 T cell subsets profile were observed (Figure A2 in Appendix). Similarly, the percentages and total number of CD3^+^ cells in peripheral blood increased until day 4 while starting to decline at day 8. This decline of peripheral T cell number was even more evident if the mice started to show signs of disease at day 8.

**Figure A3:**
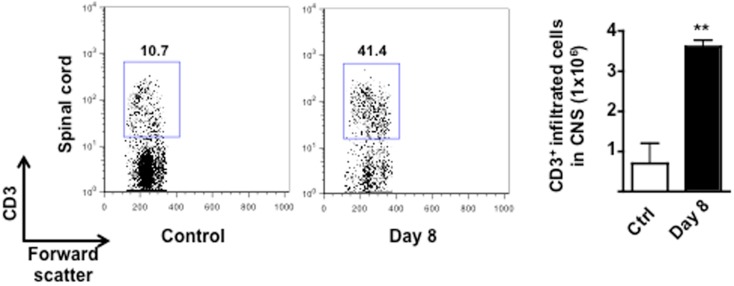
**Infiltration of T cells in the CNS of MOG_35–55_-immunized mice**. The dot plots and bar graph show the comparison of the percentages and total number of CD3^+^ T cells infiltrated in the spinal cord of control or MOG_33–55_-immunized mice. Values are expressed as mean ± SEM for three mice and are representative of *N* = 3 separate experiments. **p < 0.01 indicates significant values compared to control mice.

The reduction of circulating T cells at day 10 coincided with the expected infiltration in the CNS. Indeed, consistent with other previously published studies, very few T cells were detected in the spinal cord of control mice while a significant increase (fourfold) were found in the same tissues of the EAE mice at day 8 (Figure A3 in Appendix). The percentage of T cells further increased as the EAE progressed and was directly correlated to the severity of the disease (data not shown).

### T cell mobilization at the early stage of EAE is independent of peripheral inflammatory cytokines and not related to immunization with CFA

**Figure 4:**
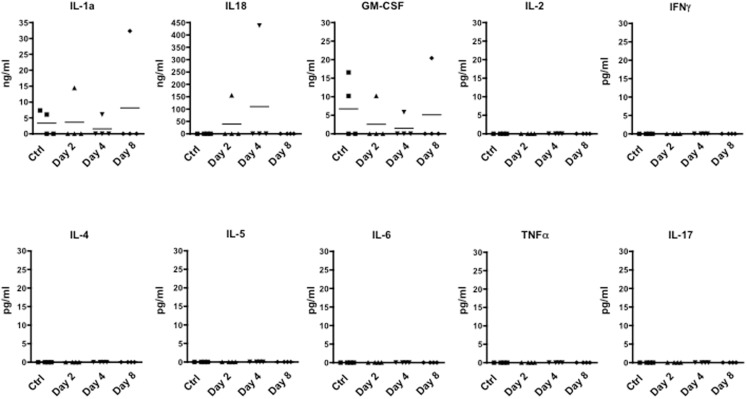
**Levels of inflammatory cytokine in the plasma of MOG_35–55_-immunized mice**. The graphs show the level of the indicated cytokines in the plasma of control or MOG_35–55_-immunized mice sacrificed at the indicated time points. The results are representative of *n* = 4 mice.

We next investigated whether the time dependent emigration of T cells in circulation and the changes in behavior we observed were due to changes in circulating inflammatory cytokines. When we scanned serum samples for inflammatory or classical T cell cytokines, only IL-1, IL-18, and GM-CSF could be detected. However, none of these mediators was differentially modulated over time (Figure 4) ruling out the possibility that none of cytokines we have measured (IL-2, IFN-γ, IL-4, IL-5, IL-6, TNF-α, and IL-17) are released in circulation upon immunization and could be associated with the changes of rearing latency.

**Figure 5:**
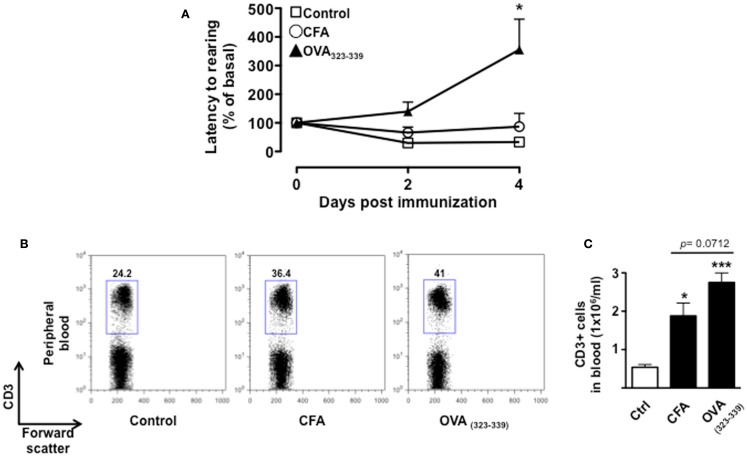
**The increase in latency to rearing relies on T cell antigenic stimulation**. The graph in **(A)** shows the percentage of latency to rearing of control, CFA only or OVA_323–339_-immunized mice. Values are expressed as mean percentage ± SEM of the basal activity for eight to nine mice. The dot plots **(B)** show the percentages of CD3^+^ T cells while the bar graph **(C)** shows the comparison of the total number of CD3^+^ T cells in peripheral blood of control, CFA only or OVA_323–339_-immunized mice at day 4. The results are representative of *n* = 6–8 mice.

To further demonstrate that the changes in behavior we observed over time were associated with T cell activation and not just the effects of CFA, we tested mice immunized with CFA only or with the immunogenic peptide OVA_323–339_. We used the change in latency as this was the parameter that gave us the highest fold changes and hence most suitable to appreciate any modulatory effect. This parameter shows the lapse in time to the first “reactive and solution-seeking” event and suggests a delay to react to unexpected and novel conditions (the open field) – a response that is typical of anxious state. As shown in Figure 5, CFA only immunized mice showed no difference compared to vehicle-injected mice while OVA_323–339_ immunized mice showed a significant (*p* < 0.05) increase in latency (Figure 5A).

Immunization with CFA only caused a significant increase in the percentage of CD3^+^ T cells that was associated with the expected leukocytosis induced by this treatment. However, mice treated in the same condition but immunized with OVA_323–339_ showed a further and significant increment in the percentages and number of circulating T cells (Figures 5B,C, respectively). Together these results provide a further link between antigenic stimulation of T cells and emotional response in mice.

### Glatiramer acetate attenuates the increased digging latency of MOG_35–55_-immunized mice

**Figure 6:**
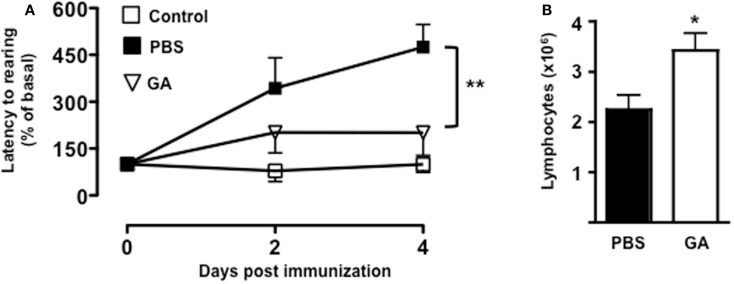
**Glatiramer acetate inhibits the emotional changes of MOG_35–55_-immunized mice**. The graph in **(A)** shows the percentage of latency to rearing of vehicle-treated non-immunized mice, glatiramer acetate-(GA), or control PBS-treated mice subjected to MOG_35–55_-induced EAE and assessed during the 5-min test. The values are expressed as mean percentage ± SEM of the basal activity for six to eight. **p < 0.01 indicates significant values compared to control mice. The bar graph in **(B)** shows the total number of T cells in the draining lymph nodes of glatiramer acetate-(GA) or control PBS-treated mice subjected to MOG_35–55_-induced EAE at day 4. The results are representative of *n* = 6–8 mice.

To further confirm the link between T cell emigration and increased latency to rearing, we pre-treated mice with a known immunomodulatory drug that is effective in the treatment of MS. As shown in Figure 6A, administration of dose of GA that inhibits the development of EAE (45) (data not shown) caused a significant reduction in the latency to rearing (*p* < 0.01). Most importantly, when we counted the number of T cells in the draining lymph nodes, we could observed a significant increase in cell number in mice treated with GA compared to those receiving PBS control (Figure 6B). This result further suggested that the retaining of T cells from the circulation significantly influence and mirror the changes in behavior.

## Discussion

Emerging evidences have shown that T cells contribute to functions other than those related to the immune response (30, 33, 46–50). The aim of this study was to explore a possible correlation between T cell activation and behavioral changes that occur at the early stage of the MOG_35–55_-induced EAE. Consistent with previous observations (24, 25, 27, 28, 40) and the results obtained by Haji et al. (27), immunized mice showed a reduced number of crossed central squares in the open field and an overall decrease in exploratory activity as indicated by the reduced number of squares.

The EAE is a classical model of autoimmune diseases where mice are immunized with an antigen that resembles a tissue component of the target organ. Activated antigen presenting cells present the antigenic MOG_35–55_ to T cells in the local draining lymph nodes (37, 42). Clonally activated T cells expand and then move first into the blood stream and thereafter into “homing licensing organs” like the lung (51). Here, their membrane make-up and gene profile change to acquire a “pathogenic” phenotype. These “licensed” cells are in fact capable of infiltrating the target organs (spinal cord and brain in this case) and initiate a cascade of events that ultimately lead to chronic inflammation and tissue damage (52).

Consistent with this model, our results show that the number of T cells increases in the spleen of immunized mice and this is followed by their migration into the bloodstream (time when the changes in emotional behavior occur) and ultimately into the CNS (time when the emotional behaviors come back to basal level). In light of these findings, it is possible to hypothesize that the two stages of T cell movement, i.e., first in the bloodstream and then into the CNS, mirror the two stages of MS development: mood changes first and motor dysfunction later. These events are not antigen specific (in this case neuronal antigen specific) or a specific feature of MS. Indeed, the results obtained using another non-endogenous antigen such as OVA_323–339_ provided us with the same findings obtained with the MOG_35–55_. This highlights the importance of T cell priming and expansion rather than a general inflammation for the emotional changes. Indeed, the lack of any significant changes in circulating cytokines suggests that these events are not part of the classical sickness behavior observed during acute inflammation (53). Further studies, now in progress in the lab, will verify this hypothesis in other models of autoimmune disease such as the collagen-induced arthritis or double strand DNA/peptide-induced lupus. The validation of these findings might indeed explain or provide a “consensus hypothesis” for the high incidence of mood disorders as a common feature of autoimmune pathologies.

Although we have not explored the passage of T cells through the lungs in our system, there are some indications that this might be a likely event. Several studies have already shown that trafficking of T lymphocytes to specific organs, such as the skin and lungs, is part of the body’s defense mechanism following acute psychological stress (35, 36, 54). It is interesting to note at this regard that patients suffering panic and anxiety attack often declare to be “out of breath” and that problems get “under our skin” when there is something that we cannot be rid of.

On a more scientific ground, seminal investigations from Schwartz’s group have recently shown that T lymphocytes migrate to the brain in response to psychological stress and that their function there is to alleviate its negative behavioral consequences. In addition to this, the authors also showed that immunization of T cells with a CNS-related peptide reduced the stress-induced anxiety and restored levels of BDNF, shown to be important for stress resilience (55, 56). In light of these findings, it is feasible to hypothesize that the drop in latency we have observed in our study might be due to the infiltration of T cells in the CNS and concomitant induction of a protective “resilient response.”

Further studies by Kipnis’s team have provided further insights on the multiple roles of T cells as homeostatic keepers of CNS functions. The authors were the first to describe a critical role for T cell derived IL-4 as key cytokine involved in learning and memory through regulation of myeloid cells present in the meningeal space (32, 57). This intriguing new concept has been recently confirmed by showing improved learning and memory in T cell deficient SCID mice adoptively transferred with M2 macrophages (58). Considering the well-known crosstalk between cognition and emotion regulation (31), it would be interesting to explore the possible changes in myeloid cell phenotype during the early stages of the EAE.

Glatiramer acetate, known in the clinic as copaxone, is one of the most common disease-modifying drugs together with interferon beta. Although its mechanisms of action have not been fully defined, a great deal of evidence suggests that it acts directly or indirectly on T cell activation (59–62). When we tested it in our system, we could clearly see a significant reduction of latency to rearing (Figure 6). Most interestingly, we also observed that GA pre-treatment caused a significant retention of T cells in the periphery compared to control mice. In light of these findings, it is tempting to speculate that the reduced activation of T cells, while reducing the signs of motor dysfunctions and the progression of the disease, it also reduces emotional changes.

In conclusion, the results of this study suggest a further mechanism (besides CNS inflammation) for the link between the neuronal and immune systems – more specifically the emotional state and immune response – during the course of autoimmune diseases like MS. The validation of these results in the clinic, together with further exploration of the mechanism by which T cells cause debilitating mood changes during the early stage of MS, might help to identify alternative immunomodulatory treatments with reduced impact on the mental well being of these patients.

## Conflict of Interest Statement

The authors declare that the research was conducted in the absence of any commercial or financial relationships that could be construed as a potential conflict of interest.

## Footnotes

^1^http://www.mssociety.org.uk/forum/everyday-living

^2^http://www.thisisms.com/forum/
